# Development of a Management Algorithm for Post-operative Pain (MAPP) after total knee and total hip replacement: study rationale and design

**DOI:** 10.1186/s13012-014-0110-3

**Published:** 2014-08-28

**Authors:** Mari Botti, Bridie Kent, Tracey Bucknall, Maxine Duke, Megan-Jane Johnstone, Julie Considine, Bernice Redley, Susan Hunter, Richard de Steiger, Marlene Holcombe, Emma Cohen

**Affiliations:** Epworth/Deakin Centre for Clinical Nursing Research, School of Nursing and Midwifery, Deakin University, 221 Burwood Highway, Burwood, 3125 VIC Australia; Plymouth University, Drake Circus, Plymouth England; School of Nursing and Midwifery, Deakin University, 221 Burwood Highway, Burwood, 3125 VIC Australia; Eastern Health/Deakin University Nursing and Midwifery Research Centre, School of Nursing and Midwifery, Deakin University, 221 Burwood Highway, Burwood, 3125 VIC Australia; Epworth Victor Smorgon Chair of Surgery, Epworth HealthCare, 185-187 Hoddle Street, Richmond, 3121 VIC Australia; Epworth HealthCare, 62 Erin Street, Richmond, 3121 VIC Australia

**Keywords:** Pain, Pain management, Post-operative pain, Algorithm, Orthopaedic, Evidence-based practice, Clinical decision making, Implementation

## Abstract

**Background:**

Evidence from clinical practice and the extant literature suggests that post-operative pain assessment and treatment is often suboptimal. Poor pain management is likely to persist until pain management practices become consistent with guidelines developed from the best available scientific evidence. This work will address the priority in healthcare of improving the quality of pain management by standardising evidence-based care processes through the incorporation of an algorithm derived from best evidence into clinical practice. In this paper, the methodology for the creation and implementation of such an algorithm that will focus, in the first instance, on patients who have undergone total hip or knee replacement is described.

**Methods:**

In partnership with clinicians, and based on best available evidence, the aim of the Management Algorithm for Post-operative Pain (MAPP) project is to develop, implement, and evaluate an algorithm designed to support pain management decision-making for patients after orthopaedic surgery. The algorithm will provide guidance for the prescription and administration of multimodal analgesics in the post-operative period, and the treatment of breakthrough pain. The MAPP project is a multisite study with one coordinating hospital and two supporting (rollout) hospitals. The design of this project is a pre-implementation-post-implementation evaluation and will be conducted over three phases. The Promoting Action on Research Implementation in Health Services (PARiHS) framework will be used to guide implementation. Outcome measurements will be taken 10 weeks post-implementation of the MAPP. The primary outcomes are: proportion of patients prescribed multimodal analgesics in accordance with the MAPP; and proportion of patients with moderate to severe pain intensity at rest. These data will be compared to the pre-implementation analgesic prescribing practices and pain outcome measures. A secondary outcome, the efficacy of the MAPP, will be measured by comparing pain intensity scores of patients where the MAPP guidelines were or were not followed.

**Discussion:**

The outcomes of this study have relevance for nursing and medical professionals as well as informing health service evaluation. In establishing a framework for the sustainable implementation and evaluation of a standardised approach to post-operative pain management, the findings have implications for clinicians and patients within multiple surgical contexts.

## Background

A major focus of the worldwide drive to improve the quality and safety of healthcare is the flexible standardisation of care processes to ensure patients receive care based on best available evidence and do not experience unnecessary variations in quality of care. Pain management after surgery is known to be a care process where the quality of care is highly variable. Prevalence studies worldwide identify that over 80% of hospitalised patients experience pain [1]-[4]. Pain in the post-operative context is often undertreated [1],[5] and is one of the main post-operative adverse outcomes [6]-[8] despite the availability of analgesics and best evidence regarding their use. In Australia, a review of research concluded that 24% to 40% of hospitalised surgical patients experienced `significant’ pain [9]. Further, a meta-analysis of published data (1973 to 1999) revealed an overall incidence of moderate to severe pain for post-surgical patients calculated at 30% (range: 26% to 33%) and severe pain at 10% (range: 8% to 13%) [10]. As a consequence of poor pain management, patients experience unnecessary suffering and higher incidence of post-operative complications [11]. More specifically, patients with poorly managed pain are at risk of developing chronic post-surgical pain [12],[13], experiencing long-lasting psychological distress [13], and acute neurohormonal changes [13].

Poor use of analgesics has been identified as a prime reason why patients experience unnecessary pain. Robust findings indicate that patients receive 50% or less of the analgesics prescribed [5],[6],[14],[15], do not receive additional analgesics in response to reports of high levels of pain [14],[15], do not benefit from the therapeutic outcomes of multimodal analgesia [16], and experience side effects of analgesics such as nausea, vomiting, constipation, sedation, or bleeding [12],[17]-[19]. In contrast, the effective use of analgesics can lead to positive outcomes for both patients and healthcare organisations, such as reductions in pain [20], opioid use [8],[21], nausea and vomiting [17], hospital length of stay [11],[22], and occurrences of unplanned hospital re-admissions [23], as well as improved mobilisation [20],[24],[25], bowel function [24], food intake [24], exercise capacity [24], health-related quality of life [24], and sleep quality [20]. Given these significant benefits of effective pain management and the potential consequences of poor analgesic control, current practices need to be consistent with guidelines developed from the best available scientific evidence.

Standardisation of care processes to improve quality and safety of patient care has the potential to transform the way healthcare is delivered. In 2001, the Institute of Medicine's document *Crossing the Quality Chasm* highlighted the imperative for patients to `…receive care based on the best available scientific knowledge,’ and that `…care should not vary illogically from clinician to clinician or from place to place’ [26]. The challenge of standardising care processes is to achieve the right balance between maintaining professional autonomy by allowing clinicians to use clinical judgement and providing clinical support systems to assist clinicians to select from, and deliver, the best treatment options. Clinical situations that hold the greatest challenge for standardising care are those that are complex, ill-structured, and have high levels of uncertainty. Pain management in the post-operative context is representative of this type of clinical situation. Despite the dissemination of best-evidence regarding pain and pain treatment there is not a structured, systematic strategy to incorporate complex clinical decision-making support systems into practice to standardise care processes and improve quality of pain management at point of care.

### Multimodal analgesia

To assist health practitioners in the delivery of effective pain management, professional groups including the Australian and New Zealand College of Anaesthetists and Faculty of Pain Medicine have published a thorough review of the scientific evidence supporting acute pain management [11] and the Procedure Specific Postoperative Pain Management Group (PROSPECT) have conducted procedure-specific systematic reviews regarding pain management [27]. Best practice recommendations for pharmacological pain management are for a multimodal approach to treatment, with patients receiving a mixture of weak and strong analgesics, administered both locally and systemically. Single analgesics alone, either opioids or non-steroidal anti-inflammatory drugs (NSAIDS), are not able to provide effective pain relief for most moderate or severe pain and are associated with unacceptable side effects [16]. The combination of drugs with different mechanisms of action produces synergistic analgesia with lower total doses of individual analgesics and fewer side effects [17]. Additionally, the timing of the administration of certain adjuvants (*e.g.*, pre-emptive) has been shown to influence the dose of analgesics required to manage post-operative pain [28].

Although current guidelines for pharmacological pain management are comprehensive, they are not presented in a form that allows healthcare staff to readily implement recommendations into everyday practice. Consequently, although multimodal analgesics are often prescribed for patients in the post-operative setting, evidence from local contexts suggests that there is substantial variation in prescribing, and analgesics are not used in a multimodal combination. Rather, they are used in a linear way in keeping with the expected trajectory of recovery after surgery so that strong analgesics (such as strong opioids) are used early post-operatively followed by non-opioids. Evidence from previous research of pain management for cancer patients highlighted the poor control of patients’ pain despite access to various analgesic treatment options [15]. Clinicians use a wide range of pain assessment and treatment approaches. In general, analgesic administration is not guided by specific policy but is instead, left to the discretion of individual medical or nursing clinicians, often with less than satisfactory pain management outcomes.

### The nature of pharmacological administration decision making in clinical practice

Nurses play a major role in post-operative pain management because they assess pain and administer prescribed analgesics across the 24-hour continuum of patients’ recovery. Decision-making related to analgesic administration involves a complex interaction between patients’ reports of pain intensity, their behavioural and physical manifestations of pain, clinician and/or patient attitudes and beliefs about pain treatment, knowledge of pain mechanisms and treatments, and clinicians’ previous experience with patients in pain. The essential principles of effective pain management are the subjectivity of pain (the need to accept patients’ interpretation of their pain intensity and experience), the selection, titration, and administration of pain relieving medicines (analgesics) and instigation of non-pharmacological pain relief strategies.

Applying these principles to individual cases within the context of the clinical work environment is complex. Clinical realities influence the management of pain in acutely ill patients. Acute pain management is complex because of the unpredictable variability of pain (incidence, intensity, and duration), patient characteristics, and pharmacological factors [11],[29]. This manifests in a variety of ways in the clinical setting. Patients often experience severe pain past the expected trajectory for a particular surgical procedure. Breakthrough pain can occur prior to the time for the next dose of analgesic necessitating decisions regarding dosage and timing of subsequent doses and adequacy of the existing prescription. Adding to the complexity of pain management are organisational factors, such as local policies, staffing levels and characteristics, workloads, time of day, availability of doctors and nurses for consultation, and workplace culture and variability that all affect day-to-day decision making. There are longstanding and repeated findings of a poor relationship between patients’ pain intensity and the amount and type of analgesic administered by nurses [5],[6],[15],[30]-[32]. These findings indicate the need to support nurses’ decision-making related to the administration of analgesics.

Previous research into the prescription practices related to analgesics has revealed that a large proportion of analgesics are prescribed to be administered as PRN (*pro re nata*, as required rather than on a fixed schedule) [15],[32] with little guidance to ensure that patients receive effective multimodal analgesia with minimal side effects [6],[15],[32]. There is variation within and between doctors’ and surgeons’ prescriptions for the type and quantity of analgesics. This adds to the complexity of day-to-day decisions by nurses about analgesic administration.

### Translation of evidence into pain management practices through the creation and implementation of a clinical support algorithm

Exploratory work has identified that analgesics are not administered according to best practice recommendations and a significant proportion of patients experience moderate to severe pain while receiving less than 50% of the analgesics they have available [6],[15],[33] or experience unnecessary side effects associated with analgesics because they are administered inadequately. There is a demonstrable need for decision support for the pharmacological management of pain to facilitate multimodal analgesia.

It is proposed that one such solution, not previously considered, is the development and implementation of a Management Algorithm for Post-operative Pain (MAPP) to support the sustainable translation of evidence-based pain management practice. The MAPP will be developed in partnership with clinicians and will address three key elements of pharmacological pain management: multimodal prescribing analgesics; assessment and management of breakthrough pain; and prevention of side effects of analgesics (Table 1). Algorithms are schematic models of the clinical decision pathway described in a guideline, presented as step-by-step methods of solving problems and are increasingly being used in healthcare to support clinical decision-making and to improve patient care [34]. As graphical representations of clinical practice guidelines, algorithms serve to promote the standardisation of clinical processes while allowing for the clinical variation that occurs in real-world contexts. In the area of pain management, algorithms have been used sparsely, *e.g.*, to assist nurses with pain management in post-anaesthetic care units [35], for post-operative intramuscular analgesia [36],[37], and for pain management following specific procedures (*e.g.*, hematopoietic cell transplants [38]) and conditions (*e.g.*, persistent shoulder pain [39], spinal cord injury [40]). None of these algorithms, however, have been designed to address the complexity of pharmacological pain management in the post-operative context where there is high variability in prescribing practices, staff skill mix, patient characteristics and risk, and a wide array of available classes of analgesics.

**Table 1:** **Key elements of the MAPP**

Element	Principles
Multimodal analgesic prescribing	Reduced variability between prescribers
	Multimodal opioid sparing regime (41,42)
	• Strong opioid
	• Weak opioid
	• NSAID*
	• Paracetamol
	Analgesics prescribed on a fixed schedule
Assessment of pain	Use of 11-point numerical rating scale for pain intensity
	Pain intensity rating ≥4 (pain or rest) indicative of breakthrough pain
	Frequency of pain intensity assessment
	• Two-hourly when awake
	• Four-hourly when asleep
	• Reassessment of pain intensity within 30 minutes of treatment for breakthrough
Management of breakthrough pain and prevention/control of analgesic side effects	Availability of analgesics prescribed as PRN
	Algorithmic support for treatment decisions
	Availability of pre-emptive treatments for nausea and constipation
	Escalation plan if breakthrough pain persists
	Review of causation and plan of treatment to prevent recurrence

*non-steroidal anti-inflammatory drugs (NSAID).

Although post-operative pain is poorly managed generally, the study will be focused in orthopaedic surgery because this surgery is common and is associated with high levels of pain that, if not well managed, interferes with rehabilitation, recovery, and long term benefits of surgery. Prolonged moderate to severe pain can lead to chronic pain in a proportion of patients [41]-[43]. The MAPP will be developed for patients undergoing total hip or knee replacement surgery because of the emerging evidence that pain management practices should be procedure specific [44]-[47]. The number of hip replacement surgeries performed in Australia since 2003 has increased by 39.2% and knee replacement surgeries have increased by 63.5% [48].

### Aims

The project will be conducted in three incremental phases to achieve the aims of the project:

1. Develop a post-operative, pharmacological pain management algorithm based on best available evidence in collaboration with a multidisciplinary panel to effectively manage pain in the post-operative orthopaedic environment (phase one);
2. Implement the pain management algorithm using an evidence-based, multidimensional framework for the successful implementation of change in practice (phase two);
3. Evaluate the impact of the algorithm on patients’ pain experience and pharmacological pain management practices across multiple sites using a pre- and post-implementation designed study (phase three).

More specifically the objectives are to:

1. Examine and calculate the proportion of post-operative analgesic prescriptions that comply with the MAPP;
2. Determine the proportion of patients who experience moderate to severe pain after TKR and THR, pre- and post-implementation of the MAPP;
3. Explore the efficacy of MAPP prescribing and administration by comparing the pain intensity ratings of patients whose analgesic prescriptions comply with MAPP recommendations with the ratings of patients whose prescriptions do not comply;
4. Examine medical records for documented rationale explaining variation to MAPP prescribing recommendations.

## Methods

### Design

There are two fundamental components of this research: the systematic development of the management algorithm for post-operative pain (MAPP) that is ecologically valid and based on best available evidence (phase one), and implementation of the MAPP into clinical practice in a way that ensures uptake and integration (phase two), and establishes a framework for measuring sustainability. Improving pain management is a multidisciplinary endeavour and is dependent on strong collaborative relationships between disciplines, administrators and patients themselves. There is potential for widespread uptake of the pain management algorithm however it is necessary, in the first instance to quantify the benefits in terms of quality improvement and develop a model for widespread implementation. The three-phase approach to this project will enable consultation and cooperation between disciplines and with patients (phase one), and identify barriers and enablers for successful implementation (phase two).

The design of phase three is a pre- and post-implementation study to evaluate the uptake and outcomes of the MAPP in three sites.

### Setting

Phases one and two will be conducted in the orthopaedic units of a large private hospital in Melbourne, Australia (coordinating site) and will be rolled-out (phase three) in two additional orthopaedic sites in Melbourne, one private hospital, and one public hospital to test transferability and uptake. Three hospitals will participate in phase three, two private sector and one public sector. More than one-half of all hip replacement surgeries are performed in the private sector (59.3% in 2011) and more than two thirds of all knee replacement procedures are performed in private hospitals (69.4% in 2011) [48].

### Phase one: development of the MAPP

The purpose of phase one is to develop the MAPP, determine the implementation strategy in the coordinating site, and plan the roll out to the two other participating sites. The first step is to identify the local context issues related to pain management and determine the scope of the guidelines. This will be achieved through a series of point prevalence surveys in the orthopaedic units to determine peri-operative, analgesic prescribing practices of anaesthetists and surgeons, and post-operative pain outcomes of patients. The second step is to convene an expert multidisciplinary panel and reach a consensus on the MAPP analgesic guidelines that is both context and procedure (total hip and total knee replacement) specific.

It is envisaged that the evidence from the point prevalence surveys conducted will assist participating anaesthetists and surgeons to reach consensus about standardising analgesic prescriptions for peri-operative orthopaedic patients within the local context.

### Prevalence surveys of pain management practices and pain outcomes

Sequential point prevalence surveys will provide essential baseline and outcome data throughout the three phases of this project. The point prevalence surveys will be conducted on the orthopaedic wards of participating hospitals. Data will be collected by research personnel who are also nurses and therefore familiar with pain assessment and analgesic prescribing.

### Participants

The study participants are all anaesthetists and surgeons who prescribe perioperative analgesics for patients undergoing THR and TKR surgery, nurses employed on the orthopaedic units who administer analgesics, and post-operative patients present on the wards at the time of the point prevalence surveys.

### Inclusion criteria (patients)

1. Aged 18 years and over.
2. Have undergone a total hip or total knee replacement in the previous 7 days (*i.e.*, are Day 0 - 7 post-surgery).
3. Are present on the orthopaedic ward at the time of the point prevalence survey(s).

### Procedure

Data collection for the surveys will include patient interviews and medical record audit. Prevalence of pain, pain intensity, and the impact of pain on activities of daily living will be collected via brief interviews with patients (approximately five minutes) about their pain experience during the previous 24 hours. In order to capture the prescribing practices of all anaesthetists and surgeons, and the pain outcomes of patients across the recovery trajectory (Day 0 - 7), five prevalence surveys will be conducted over five weeks. In week one, the surveys will be conducted on Monday, in week two on Tuesday, and so on. In the coordinating centre, there are expected to be approximately 60 eligible patients available per day.

### Patient interviews

1. patients’ demographic characteristics and relevant medical history.
2. Pain intensity rating of current pain (pain at rest) and worst and average pain in the previous 24 hours will be measured on an 11-point numerical rating scale (NRS) where 0 denotes no pain and 10 denotes worst pain possible) [5].
3. Interference with activities of daily living and patient satisfaction will be measured using sections of the revised American Pain Society Patient Outcomes Questionnaire (APS-POQ-R) [49],[50], a well-validated instrument that has been used in a number of studies [5].

### Medical record audit

#### Pharmacological pain treatment survey

This survey, used previously by the investigators, elicits patient information relating to height, weight and body mass index, current surgery and sequelae, as well as the type and quantity of analgesics prescribed prior to admission, the type of anaesthetic techniques used intra-operatively and the type and quantity of analgesic and symptom management medications prescribed and administered in the 24-hour period prior to the patient interview.

### Data analysis

The purpose of the point prevalence survey in phase one is to inform the development of the MAPP by providing rich descriptive data of current analgesic prescribing and administration practices to allow comparisons with guidelines based on best available evidence. patients’ pain outcomes will provide evidence for the need to change existing practices and identify where the main gaps in practice are, for example whether the gaps relate to prescribing of analgesics or treatment of breakthrough pain. In addition, the survey data will be the pre-implementation data for evaluating the success of the implementation of MAPP in phase two.

Frequencies and other descriptive statistics will be used to describe the sample, pain intensity ratings and impact of pain on activities of daily living. Pain intensity ratings greater than 3 and 7 or less, will be categorised as moderate pain and ratings greater than or equal to 8 will be categorised as severe pain. Analgesic prescriptions will be described in terms of drug group (strong opioid, weak opioid, non-steroidal anti-inflammatory drug (NSAID), or paracetamol) and available doses over 24 hours. The amount and type of analgesics patients receive will be analysed as a ratio of the total amount prescribed for the 24-hour period prior to the survey. In addition, The *Pain Management Index* will be calculated for each patient. Scores are calculated based on the relationship between worse pain intensity scores and potency of analgesic administered. Evidence of the validity of this index has been demonstrated by Ward et al. in the cancer pain management context [51].

### Expert multidisciplinary advisory panel

Following the point prevalence surveys, an expert advisory panel will be convened with multidisciplinary and consumer representation to advise on the development, dissemination, and implementation of the guidelines. We will use the pre-intervention survey results to highlight current practice and engage clinicians for improving post-operative pain management and consequently improving pain outcomes for patients. The panel will include the project team with the addition of representatives from the craft groups of surgeons, anaesthetists, pharmacists, nurses and patients. A representative from health information services will advise on the integration of the algorithm into permanent medical records. Key members of the panel (surgeons, anaesthetists) will also liaise with their colleagues to promote standardisation of prescribing practices.

The brief for the multidisciplinary panel at the coordinating site will be to assist the development of a MAPP based on the best scientific evidence and focused on standardising analgesic prescription and able to be applied by registered nurses caring for post-operative orthopaedic patients who have undergone total knee replacement (TKR) or total hip replacement (THR) surgery.

To develop the pharmacological pain management algorithm, we will follow the general steps of guideline development published by the National Health and Medical Research Council [52]. The panel at the coordinating site will evaluate best available evidence, management of risk, local practice issues that may impact upon uptake and utilisation of the algorithm, transferability of the algorithm to other orthopaedic post-operative environments and evaluation of the outcomes post-implementation. The panel will also consider the natural history of the conditions being treated, the possible outcomes of MAPP, the benefits versus the risks of each intervention, and the clinical supports required to facilitate safe uptake of the intervention. The panel will advise on the dissemination and implementation of the MAPP at the coordinating site and also consider the specific modifications for its application at the two roll out sites.

A Clinical Nurse Consultant (CNC) role will be also be created. The CNC will act as the project facilitator in the development, implementation and evaluation of the pharmacological algorithm. The CNC will be based at the coordinating institution and have significant experience in post-surgical nursing and pain management and also be able to play a significant role in the expert panel. The CNC will also be required to liaise with the interdisciplinary panel members and orthopaedic surgeons and anaesthetists, and be involved in the development of the algorithm.

Consideration will be given to presenting the algorithm in a form that facilitates comprehension by clinicians and is flexible to suit information needs and knowledge levels of different audiences (*e.g.*, consumers, specialist and non-specialist health professionals) and one that complies with national and local criteria regarding the prescription of medications.

### Phase two: development and pilot of the implementation framework for the MAPP

The aim of phase two is to formulate the dissemination, implementation, revision, and evaluation strategies. This phase of implementation of the MAPP will occur in the coordinating site to identify barriers to uptake and adherence, and to refine the implementation strategy for the rollout to the other two sites.

### Implementation framework

The Promoting Action on Research Implementation in Health Services (PARiHS) framework [53]-[55] will be used to guide the implementation of the clinical algorithm into practice. Research on the diffusion or adoption of innovations suggests that successful change implementation is a function of the relationship between evidence, context, and facilitation [54]. The PARiHS framework has been used successfully in an action research study on improving post-operative pain management [56]. In partnership with key clinicians and clinician groups and other stakeholders we intend to implement and evaluate the pain management algorithm using this framework. For implementation to be successful, there needs to be clarity about the nature of the evidence being used, the quality of the context, and the type of facilitation needed to ensure a successful change process. These steps are outlined in detail below.

### Evidence

According to the PARiHS framework [53]-[55], evidence comes from four sources: research, clinical experience, patient experience, and local data/information. In the MAPP study, research evidence will be derived predominantly from the acute pain management guidelines [11] and consensus from the members of the expert panel; patient experience will be informed by patient representation on the panel, and local data/information will come from the prevalence surveys and the resources of the panellists who are in clinical positions.

### Context

Context*,* according to the PARiHS framework, is made up of three factors: culture, leadership, and evaluation. Brown and McCormack [56] contend that learning cultures, transformational leadership, and effective evaluation mechanisms promote the implementation of research into practice. Context is a core component of the implementation design of the MAPP. The appointed Clinical Nurse Consultant (coordinating site) and Clinical Nurse Specialists at the roll out sites will oversee the pilot implementation framework. Ongoing evaluation mechanisms will provide feedback to promote the development of a model for the successful implementation of MAPP into practice.

### Facilitation

Facilitation refers to the process of enabling or making easier the implementation of change and is achieved by individuals carrying out specific roles within organisations to facilitate change [42]. We will use strategies that have been consistently effective in producing evidence-based practice changes [52],[57], including interactive educational meetings (*e.g.*, active participation in workshops, small-group discussion, problem-based learning), educational outreach visits (*e.g.*, trained personnel providing face-to-face visits to clinicians), and other reminders (*e.g.*, visual resources and reminders about using the algorithm). Research personnel in the project team will oversee the education component and develop an on-line education module for the multi-site roll out.

### Prevalence survey of pain management practices and pain outcomes: pilot implementation

To evaluate the uptake and efficacy of the algorithm, another survey of pain management practices and pain outcomes will be performed 10 weeks after the implementation of the MAPP in the orthopaedic units. The results will be compared to the pre-implementation survey. The same procedure will be used to collect these data.

The extent to which clinicians have adopted the pain management algorithm in terms of prescribing and multimodal medication administration, will be determined through the medical record audit. This method will be evaluated and an ongoing evaluation strategy for continuing quality assurance and sustainability will be established.

### Primary outcomes

The primary outcome measures to determine successful implementation are the proportion of patients who have multimodal analgesic prescriptions in accordance with the MAPP, 10 weeks after implementation, to determine the extent to which clinicians have adopted the prescribing guidelines, and the proportion of patients with moderate to severe pain intensity scores to determine whether the treatment of breakthrough pain is in accordance with the MAPP.

### Sample size

The sample size calculations are based on pilot data of pain prevalence and pain management practices conducted by Botti *et al.* The minimum sample size required to detect a 20% reduction in the proportion of THR patients with moderate to severe pain intensity (current prevalence 33%, reduce to 13%) at rest is 78 and for TKR patients (current prevalence 53%, reduce to 33%) is 109. This number of participants would provide the power to show an advantage for the post-intervention group with 85% power at the 5% significance level. Therefore a minimum of 187 patients will be recruited in phase one and 187 in phase two (total n =374).

### Data analyses

Data gained from the post-implementation surveys will be compared to the pre-implementation survey data in order to determine the effectiveness of the implementation strategies for introducing the MAPP into practice. T-tests and chi-square tests will be used to compare baseline characteristics between the pre- and post-implementation patient groups. Non-parametric statistical analyses will be used to detect significant differences in the proportions of patients with analgesic prescriptions in accordance with the MAPP, and patients with moderate to severe pain. Tests will be two-tailed with statistical significance set at an alpha level of 0.05. The analyses will be undertaken using STATA^©^.

### Phase three: external application of the MAPP and implementation framework

The aim of phase three is to implement and evaluate the transferability and uptake of the MAPP and establish a framework for measuring sustainability of the MAPP in multiple sites beyond the development site. This will be an important aspect of ensuring that the MAPP fits the different contexts of public and private healthcare and is transferable with modifications for the local contexts.

There will be a 10-week, pre-implementation stage and a 10-week, post-implementation stage at each of the two rollout sites (one private and one public hospital in metropolitan Melbourne). Point prevalence surveys and multidisciplinary panel methodologies used in phase one and the implementation and evaluation methodologies applied in phase two will be repeated at each rollout site.

### Prevalence surveys pre- and post-implementation of the MAPP

The point prevalence surveys will be used to determine the quality of pain management practices at each site pre-implementation, in order to identify any site specific issues to be considered, allow modification of the implementation framework, and provide data for the pre- and post-implementation comparisons. Data collection methods including patient eligibility, survey and medical record audit will be the same as those described in phases one and two.

### Multidisciplinary panel at rollout sites to facilitate transfer of MAPP according to local contexts

A site specific multidisciplinary panel will be convened after the pre-intervention point prevalence surveys have been completed for the purpose of reviewing the findings and assessing analgesic prescribing and administration practices to determine whether there are local contextual factors that require the MAPP to be modified. The panel will oversee and advise on the contextual modification of the algorithm based on the best scientific evidence. In doing so the panel will also consider the clinical supports required to facilitate safe uptake of the intervention in each site. The CNC at the coordinating site will also work closely with the Clinical Nurse Specialists on the orthopaedic wards of the two rollout sites to facilitate implementation and evaluation of the MAPP.

### Implementation, revision, and evaluation of the MAPP at each rollout site

The implementation, revision and evaluation methodologies described in phase two in the coordinating site will be used in the rollout sites.

### Primary outcomes

The primary outcome measures identified in phase two will be applied again in phase 3 to establish the uptake of the MAPP by examining the proportion of patients whose analgesic prescriptions comply with the MAPP and the proportion of patients with moderate to severe pain at rest.

### Secondary outcome

The secondary outcome measure will be the efficacy of MAPP prescribing and administration by comparing the pain intensity ratings (current, worst, and average pain) of patients whose analgesic prescriptions comply with MAPP recommendations with the ratings of patients whose prescriptions do not comply.

In addition, process evaluation will include an examination of all medical records for any documented rationale by clinicians explaining a variation to MAPP prescribing recommendations such as allergies or contraindications.

The number and nature of site-specific changes made in order to implement the MAPP at each site will be recorded and analysed in order to inform the transferability of the MAPP across health services.

### Sample size

The phase three study is powered to detect changes in point prevalence surveys before and after MAPP implementation for the two primary outcomes. A type I error of 0.05, power of 0.85 and intra-cluster correlation effect of 0.2 to account for hospital clustering effect have been considered. Based on these assumptions and a prevalence of moderate to severe pain at rest of 33.8% for THR patients and 53.1% for TKR patients, 94 THR patients and 131 TKR patients in each prevalence survey is needed to detect a 20% reduction in the proportion of patients with moderate to severe pain at rest (Rest pain >3/10). With a total 450 patients and an estimated 15.5% compliance with multimodal analgesic prescribing (according to pilot data at the coordinating site), there is 0.85 power to detect a minimum of 13% improvement in prescribing adherence.

### Data analysis

Again pre- and post-implementation data will be compared to determine the uptake of the MAPP by doctors and nurses. T-tests and chi-square tests will be used to compare baseline characteristics between the pre- and post-implementation patient groups. Non-parametric statistical analyses will be used to detect significant differences in the proportions of patients with analgesic prescriptions in accordance with the MAPP, and patients with moderate to severe pain. Due to intra correlation of hospitals adjusting for clustering effects is necessary. Appropriate generalised linear mixed models (GLMMs) for binary outcomes with logistic link will be used to evaluate the relationships between the main outcomes and the MAPP while adjusting for any important risk factors or covariates. Tests will be two-tailed with statistical significance set at an alpha level of 0.05. The analyses will be undertaken using STATA©.

A secondary outcome to be analysed is the efficacy of the MAPP in reducing pain intensity ratings by comparing pain intensity ratings of patients where the MAPP guidelines are followed with those where the guidelines are not followed. Given that we are expecting to improve prescribing adherence from 15.5% to at least 28% (but it is likely to be much higher) we estimate we would have at least 70 in the compliance group and 180 in the noncompliance group. The minimum sample size required to measure a difference in mean pain intensity prior to and following implementation is 86. Data from previous research by Botti et al. were used to calculate power to show an advantage of at least 15 units (1.5 on VAS) for the post-intervention group with 80% power at the 5% significance level. Change differences of two points in cancer populations [58] and 3 points in surgical populations [59] have been shown to have clinical significance on a 0-10 Visual Analogue Scale.

### Ethics approval

All phases of this study have been approved by the Human Research and Ethics Committees at the coordinating hospital and two supporting hospitals. The project has also been reviewed and approved by the Deakin University Human Research Ethics Committee.

## Discussion and conclusions

One of the key priority goals of research in the area of preventative healthcare is to reduce the risk of adverse outcomes. Ineffective pain management has significant moral, clinical, and financial implications for health. The impact of poor pain management is substantial in both human and economic terms. The use of medicines is the most common method of healthcare treatment [60]. There is growing recognition that unrelieved pain can adversely affect outcomes from surgery and may lead to persistent (chronic), long term pain, with associated economic and societal costs [11]. This work directly addresses the need for evidence-based preventative interventions that reduce the incidence and severity of adverse outcomes associated with ineffective pain management.

The drive for quality improvement through evidence-based healthcare is a dominant theme in practice, management and education in health services worldwide. There is recognition of the need for system change to support the delivery of safe, high-quality care. The algorithm will be developed according to previously described recommendations for post-operative pain management for patients undergoing total hip or total knee replacement surgery. It is not enough, however, to introduce tools and strategies with which to improve quality of care without acknowledging the complexities of change processes, the need to engage staff, and the imperative to make any practice changes sustainable in the long term [61]. Traditionally, improvements in the processes of healthcare delivery in acute care occur in uncontrolled environments without the ability to adequately evaluate the outcomes. This has sometimes resulted in unintended or negative consequences occurring as a result of a change in practice. This project will evaluate the implementation of a process to improve the quality of pain management through standardising evidence-based care processes thereby assisting complex decision making by clinicians at the point-of-care. The project will also identify the key components of successful adoption across sites and healthcare sectors.

The proposed intervention directly addresses the need for evidence-based interventions that reduce the incidence of adverse events and improves the quality of care delivery. Specifically, this project addresses the high prevalence of moderate and severe pain experienced by post-surgery orthopaedic patients. Developing improved processes for the effective prescription and administration of post-operative analgesics applies not only to orthopaedic surgery but to many other surgical contexts and thus will impact on a large number of patients in Australia and internationally.

## Authors' contribution

MB conceived the study, validated the methods and was involved in drafting and approving the manuscript. BK, TB, MJJ, and MD contributed to the design of phase two. JC and BR contributed to the development of the MAPP and will oversee the development of the education modules for nurses. RdS is the convener of the multi-disciplinary expert panel and MH is the coordinator of the panel. SH and EC are overseeing the prevalence surveys and analyses. All authors have read and approved the final manuscript.

## Authors' information

1. Alfred Deakin Professor Mari Botti
  Chair in Nursing, Epworth-Deakin Centre for Clinical Nursing Research
  Deakin University
2. Professor Bridie Kent
  Plymouth University
3. Professor Tracey Bucknall
  Professor of Nursing Deakin University, Foundational Chair in Nursing, Alfred Health
4. Professor Megan-Jane Johnstone
  Professor of Nursing, School of Nursing and Midwifery, Deakin University
5. Professor Maxine Duke
  Head, School of Nursing and Midwifery, Deakin University
6. Professor Julie Considine
  Chair in Nursing Eastern Health-Deakin University Nursing and Midwifery Research Centre
7. Associate Professor Bernice Redley
  Senior Research Fellow, Epworth-Deakin Centre for Clinical Nursing Research
8. Professor Richard de Steiger,
  Epworth Victor Smorgon Chair of Surgery, The University of Melbourne
9. Ms Marlene Holcombe
  Registered Nurse, Special Projects Officer
10. Ms Susan Hunter
  PhD Candidate, School of Nursing and Midwifery, Deakin University
11. Dr Emma Cohen
  Research Fellow, Epworth-Deakin Centre for Clinical Nursing Research

## Abbreviations

MAPP: Management Algorithm for Post-operative Pain
TKR: Total knee replacement
THR: Total hip replacement
NSAID: Non-steroidal anti-inflammatory drugs
